# Liquid–Liquid Phase Separation: Mechanisms, Roles, and Implications in Cellular Function and Disease

**DOI:** 10.1096/fba.2025-00140

**Published:** 2025-11-19

**Authors:** Dikesh Kumar Thakur, Sonal Padole, Tapati Sarkar, Somasundaram Arumugam, Shiladitya Chattopadhyay

**Affiliations:** ^1^ Department of Biopharmaceuticals National Institute of Pharmaceutical Education and Research Kolkata (NIPER Kolkata) Kolkata India; ^2^ Department of Biological Sciences Vanderbilt University School of Medicine Nashville Tennessee USA; ^3^ Department of Pharmacology and Toxicology National Institute of Pharmaceutical Education and Research Kolkata (NIPER Kolkata) Kolkata India

**Keywords:** biomolecular condensates, cancer, liquid–liquid phase separation, Membraneless organelles, methods to study LLPS, neurodegenerative diseases, virus

## Abstract

Liquid–liquid phase separation is a basic biophysical process that creates essential membraneless organelles that support different cellular activities, including chromatin organization and gene expression. The malfunction of liquid–liquid phase separation (LLPS) plays a critical role in numerous diseases, such as neurodegenerative disorders, including amyotrophic lateral sclerosis (ALS) and Alzheimer's disease (AD), which involve TDP‐43 and Tau, various cancers that utilize SPOP and YAP/TAZ proteins, and viral infections where pathogens use LLPS to replicate and avoid immune detection. This review brings together the fast‐growing knowledge about LLPS across multiple scientific fields. The paper examines the physiological functions of LLPS along with its disease pathogenesis mechanisms and presents various experimental techniques (e.g., advanced microscopy, FRAP, FCS) for its investigation. It introduces new therapeutic approaches such as PTM modulation, small molecules like 1,6‐hexanediol and Lipoamide, and advanced genetic tools including CRISPR and PROTACs like PSETAC, which also explores diagnostic applications. The thorough integration of knowledge presented here is essential to connect separate scientific findings while propelling research forward and turning LLPS discoveries into new biomedical developments.

## Graphical abstract

Liquid–liquid phase separation (LLPS) is a fundamental biophysical process responsible for forming membraneless organelles involved in key cellular functions like chromatin organization and gene expression. Dysregulation of LLPS contributes to various diseases, including neurodegenerative disorders.

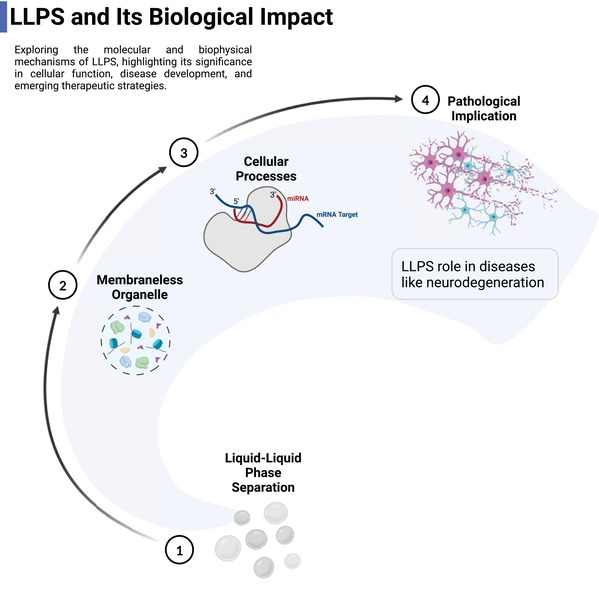

## 1 Background

Liquid–liquid phase separation (LLPS) is the spontaneous demixing of biomolecules into dense liquid‐like phases, which underlies the formation of membraneless organelles (also called biomolecular condensates or droplets) in cells [1]. In these condensates, proteins, nucleic acids, and other factors are concentrated to a high density, with correspondingly slower diffusion than in the surrounding cytosol or nucleoplasm [1]. This compartmentalization allows biochemical reactions to proceed with enhanced efficiency or specificity, effectively increasing reaction rates by colocalizing enzymes and substrates [1]. LLPS is now accepted as a general organizing principle in cell biology: Multivalent macromolecular interactions (e.g., via modular binding domains or intrinsically disordered regions, IDRs) can trigger a sharp transition when component concentration exceeds a threshold, driving the assembly of a separate liquid phase [1].

Phase‐separated condensates pervade both cytoplasm and nucleus. In the cytoplasm, LLPS governs the assembly of ribonucleoprotein (RNP) granules and signaling clusters; examples include stress granules and processing bodies (P‐bodies) that dynamically sequester mRNAs under stress, as well as specialized germline granules or spindle‐associated condensates [2]. In the nucleus, LLPS organizes nucleoli, Cajal bodies, chromatin domains, and transcriptional hubs. For instance, super‐resolution imaging revealed that RNA polymerase II and its transcription factors form liquid‐like clusters at active promoters and super‐enhancers [3]. Similarly, nuclear bodies such as Cajal bodies and speckles arise from phase separation of ribonucleoprotein factors and long noncoding RNAs (e.g., NEAT1 in paraspeckles), creating distinct liquid subcompartments. These condensates are enriched in proteins with IDRs and in RNA, reflecting the two main classes of multivalent interactions in LLPS: conventional scaffold interactions (protein–protein, protein–RNA, RNA–RNA) and weak, transient contacts among low complexity or disordered segments (π–π, electrostatic, cation–π).

Several critical molecular features induce LLPS. Intrinsically disordered protein regions (IDRs) and modular interaction domains (SH3, PRM, RNA‐binding motifs) provide multivalency that promotes networked interactions. Often, polyionic polymers (such as long RNA or DNA segments) serve as scaffolds that nucleate condensates via electrostatic or π‐based interactions. Phase separation is highly concentration‐dependent and sensitive to the cellular environment: changes in pH, temperature, salt, molecular crowding, and posttranslational modifications (phosphorylation, methylation) can dramatically tune condensate formation or dissolution.

Both experimental and computational methods are essential to study LLPS. Bioinformatic tools predict phase separation propensity based on sequence features. For example, the D2P2 database curates disorder and domain features that can flag LLPS‐prone proteins, while specialized databases (LLPSDB, PhaSePro, PhaSepDB, DrLLPS) compile experimentally validated condensate systems and their conditions [4]. Imaging and biophysical techniques are then used to detect and characterize condensates. Importantly, the dynamics and material properties of condensates are measured by fluorescence recovery after photobleaching (FRAP), fluorescence correlation spectroscopy (FCS) or related methods. In FRAP experiments, a fluorescently labeled condensate is bleached by a laser, and the rate of fluorescence recovery reports the exchange rate of molecules with the surroundings—the faster the recovery, the more liquid‐like and dynamic the condensate. Overall, this arsenal of tools—both predictive databases and laboratory techniques—has rapidly expanded the LLPS research toolkit.

LLPS underpins diverse physiological processes. Condensates act as reaction crucibles and regulatory hubs: They can locally concentrate enzymes or sequester inhibitors, control nucleic acid transactions, and sort molecules for transport. In gene regulation, LLPS helps organize transcription. For example, clusters of transcription factors, coactivators, and RNA polymerase II at super‐enhancers form phase‐separated bodies that amplify gene expression. RNP granules in the cytoplasm mediate mRNA metabolism: stress granules halt translation of nonessential mRNAs under stress, while P‐bodies participate in mRNA decay. Phase separation also plays roles in signaling and metabolism: immune sensors like cGAS form liquid condensates with DNA, which function as “reaction crucibles” to boost second‐messenger production and antiviral signaling. Likewise, synaptic proteins undergo LLPS to cluster neurotransmitter release machinery at pre‐ and postsynaptic densities. In each case, the dynamic assembly and disassembly of condensates provide a reversible mechanism to regulate cell physiology.

Aberrant LLPS is increasingly linked to disease, and LLPS itself offers new diagnostic and therapeutic avenues. Pathological protein aggregation in neurodegeneration (e.g., Alzheimer's tau tangles, TDP‐43 in ALS) may arise from dysregulated phase transitions of normally liquid‐like RNP granule components. In cancer, mutations or overexpression of transcriptional condensate scaffold proteins (such as those at super‐enhancers) can drive oncogenic gene expression programs. Viral infections can hijack LLPS: for instance, the SARS‐CoV‐2 nucleocapsid protein undergoes LLPS with viral RNA to form condensates critical for genome packaging and immune evasion. Recognizing the role of LLPS in disease has spurred interest in targeting condensates therapeutically. Early efforts suggest that chemical or genetic perturbation of condensate formation can modulate pathological pathways—for example, altering immune signaling condensates has been proposed as a novel intervention strategy. Thus, LLPS is not only a key factor in human health and disease, but also a potential biomarker and drug target, as highlighted by the rapid emergence of LLPS‐focused drug screens and diagnostic assays.

In light of these developments, a comprehensive and up‐to‐date synthesis of LLPS biology is warranted. This review will examine both cytoplasmic and nuclear condensates, the molecular drivers and physical mechanisms of phase separation, and the experimental and computational methods used to study them. We will discuss current LLPS databases and predictive tools, survey the physiological roles of condensates in processes from transcription to signaling, and analyze emerging links between LLPS and human diseases, including prospects for therapeutic or diagnostic exploitation. Given that LLPS research has expanded explosively in recent years and that its findings span multiple disciplines, an integrated overview is timely. Notwithstanding the many advances, critical gaps remain—for example, the “molecular grammar” that dictates which sequences phase‐separate is still incompletely understood, and the consequences of condensate dynamics in vivo are only partly characterized. By highlighting recent progress and unresolved questions, this review aimed to guide future work in this rapidly evolving field.

## 2 Critical Components in Inducing LLPS

There are numerous significant players. Liquid droplets can be formed by the interaction and phase separation of proteins with modular domains, such as those between the SH3 domain and proline‐rich motifs (PRMs) [5]. The capacity to regulate phase separation is determined by affinity and valency, or the quantity of interacting modules [6]. Multivalent intermolecular interactions can also be seen in proteins that have intrinsically disordered regions (IDRs), yet repeat sequences [7]. It has been noted that charged and aromatic amino acids play a significant role in the interactions between these IDRs [8]. These membraneless condensates can contain nucleic acids, particularly RNAs, which have been demonstrated to enhance LLPS induced by interactions between IDRs [9].

## 3 Mechanism of LLPS Formation

LLPS refers to a reversible physicochemical phenomenon where larger molecular components come together to form a concentrated phase alongside a less dense phase. Multiple theories explain how LLPS happens. There are many molecules suspended in the cellular domain, but when the interacting molecules come in contact with a specific organizing platform, like proteins [10, 11], RNAs [12, 13], poly ADP ribose [14], DNA [15, 16], or chromatin fibrils [16], which contain distinct binding sites for macromolecules that facilitate the formation of phase condensate, then only the interacting molecules' concentration reaches the threshold value, resulting in the formation of LLPS. The condensate is believed to be a round droplet by coalescence and quickly exchanges components with the external environment [17, 18]. Polymer–polymer phase separation is another compartmentalization technique, particularly seen in the nucleus. PPPS involves the formation of link bridges between interacting polymers [19, 20, 21]. The interaction among different biomolecules and the organizing platform for forming LLPS is noncovalent in nature, involving electrostatic, pi–pi, cation–pi, hydrogen bonding, dipole–dipole interactions, van der Waals, etc. [22, 23].

**FIGURE 1:**
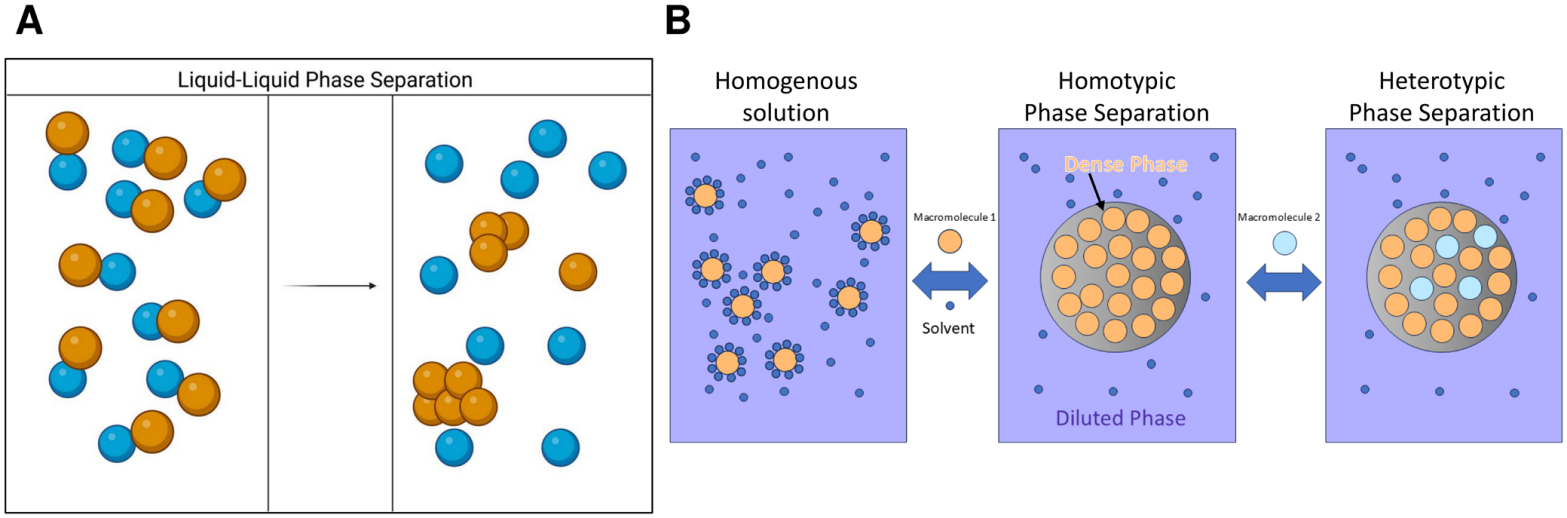
(A) Molecular self‐assembly and phase separation: In the initial state, a homogeneous solution exists where blue and orange macromolecules are uniformly distributed. Upon the introduction of specific modulatory factors, such as changes in pH, temperature, or external stimuli, the system undergoes phase separation. This results in distinct liquid‐like phases, each enriched with blue or orange macromolecules, depicting phase separation. (B) Homotypic and heterotypic interactions driving phase separation of macromolecules: Two primary phase separation mechanisms can be observed. The first is homotypic phase separation, where molecules of the same type (e.g., blue or orange) preferentially interact, leading to their clustering and formation of distinct phases. And another is heterotypic phase separation, where molecules of different types (blue and orange) exhibit a higher affinity for each other compared with their self‐association. This results in the formation of mixed phases where both types of molecules coexist and form condensates.

**FIGURE 2:**
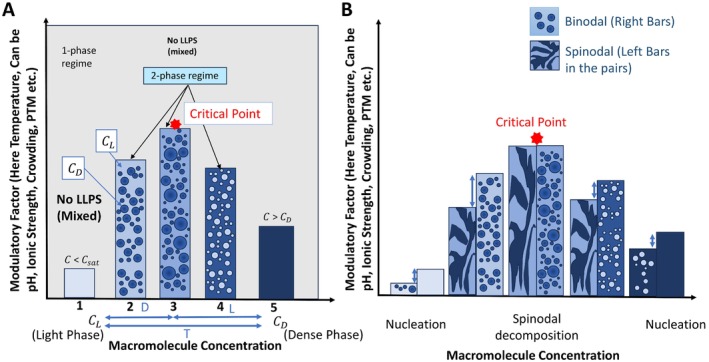
Understanding the formation of LLPS as a function of the modulatory factors: (A) Figure illustrates the phase behavior of a system as a function of macromolecule concentration and the influence of modulatory factors (e.g., pH, temperature, ionic strength). When concentration is below saturation C_sat_, or above Dense Phase concentration C_D_, the system shows no demixing and behaves in one phase, as shown in Bars 1 and 5, respectively. Bars 2, 3, and 4 represent some random points within the range of concentration of macromolecules above C_sat_ but below C_D_. Here, in the presence of specific modulatory factors, the system is demixed by separating the system into a light concentrated phase (CL) and a dense concentrated phase (CD), forming membraneless condensates. At these three points (2,3,4), the ratio of concentration of C_L_ and C_D_ is equal, but the volume is different. The critical point is the point above which LLPS formation cannot occur. (B) The spinodal bar represents the region of instability where the demixing takes place via spinodal decomposition. The area between the spinodal and binodal bar (represented by blue arrow) is where the system becomes fully nucleated, that is, where LLPS are formed.

When phase separation occurs, interactions between macromolecules or solutes are more energetically favorable than between macromolecules and solutes. This results in a gain in free energy that outweighs the entropic drive toward a homogeneous solution [24, 25]. At this point, a free energy minimum is achieved, and the two phases, despite having different solute concentrations, share the same Gibbs free energy [26]. To understand phase separation in a specific system, a phase diagram can be created by varying conditions like temperature, salt concentration, pH, or macromolecular concentration (See Figures 1 and 2). This diagram helps identify conditions that favor phase separation and assess their likelihood under different physiological conditions [27].

Another believed and observed theory is that, in specific cellular circumstances, such as posttranslational modifications, oligomerization, or nucleic acid binding, proteins can accumulate to the required concentration for liquid–liquid phase separation (LLPS). Posttranslational modifications (PTMs) like phosphorylation can modulate LLPS. For example, phosphorylation of heterochromatin protein HP1α promotes its phase separation by enabling oligomerization, while acetylation of histones antagonizes chromatin LLPS, dissolving condensates and altering chromatin states [28, 29]. Environmental variables such as temperature, pH, and ionic strength also affect phase separation dynamics. They can also trigger the formation or dissolution of membraneless organelles, allowing cells to adapt to different conditions [30].

Scaffold proteins are essential players in liquid–liquid phase separation (LLPS) and the formation of membraneless organelles (MLOs). These have numerous interaction domains that can bind to different biomolecules such as proteins, nucleic acids, and lipids. This ability to interact with multiple molecules promotes the aggregation of these molecules, creating concentrated condensates. They serve as nucleation sites for LLPS, where other molecules can accumulate and undergo phase separation. By concentrating on specific biomolecules, scaffold proteins lower the energy barrier for phase separation, enhancing the likelihood of condensate formation. Later, it organizes cellular components by establishing a framework for assembling various biomolecules. This organization is crucial for properly functioning membraneless organelles as it ensures that the right components are in the right place at the right time. By concentrating specific substrates and enzymes within a condensate, scaffold proteins can enhance the efficiency of biochemical reactions [31].

## 4 Methods to Study LLPS

### 4.1 Differential Interference Contrast (DIC) Microscope

The imaging method known as differential interference contrast (DIC) creates an amplitude difference image from a specimen's optical path gradient [32]. The polarizer, analyzer, and Wollaston prism are necessary for a DIC microscope [32]. The study of the characteristics, activities, and dynamics of LLPS in vitro frequently makes use of DIC microscopes because they provide quick and easy imaging of phase‐separated droplets generated in vitro [33]. The DIC observation of salt‐dependent LLPS implies that the primary component responsible for generating LLPS in the given system was the increased hydrophobic interactions [34]. By changing the concentration of DEX, one can modify the LLPS of amyloid‐β [34]. It was determined that DEX was more effective than PEG at promoting the LLPS of amyloid‐β because PEG did not cause LLPS under the same concentration conditions [34]. This implies that variations in the coexisting polymers' molecular weights and other characteristics could contribute to amyloid‐β's LLPS, which is known to impair the stability of biomolecules [34]. These findings also showed that as the concentration of DEX increased, the size of the aggregates formed in the solution decreased [34].

### 4.2 Wide‐Field Fluorescence Microscope and Confocal Microscope

To image samples, it uses fluorescent tags or autofluorescence [32]. It allows for the evaluation of tissues or cells that have had fluorescent markers explicitly applied for that purpose [32]. Previous studies have examined the development or characteristics of LLPS droplets in vivo and in vitro using fluorescence wide‐field microscopy [35]. For the investigation of LLPS, fluorescence microscopy offers several benefits, such as excellent repeatability in vitro, great controllability, and customizable ambient conditions [32]. Widefield optical microscopy analysis of the BSA droplet phase as a function of the amount of proline revealed that droplet size reduced as proline concentration increased [36]. It has also been noted that proline concentration increases with decreasing FUS267 droplet size [36]. The focused beam takes the place of the wide‐field beam as the excitation light source in confocal microscopy [32]. Confocal microscopy provides an efficient way to observe the phase‐separated droplets that tau protein forms in vivo or in vitro [33]. Confocal microscopy can segment biological samples finely and create high‐resolution pictures [32]. Because of its slow capture speed, it is not appropriate for capturing very dynamic processes [32].

### 4.3 Total Internal Reflection Fluorescence (TIRF) Microscopy

In cell biology, total internal reflection fluorescence microscopy, or TIRF‐M, is a commonly used optical technique that is especially useful for examining the localization and dynamics of single molecules as well as for analyzing cell membranes. In a limited sample region just next to the interface of two media with differing refractive indices, TIRF‐M can create an evanescent wave or field, with the excitation depth being less than 100 nm from the solid surface's thickness [32]. Maruri‐Lopez et al. investigated the LLPS of the single‐stranded DNA‐binding protein (EcSSB) of *Escherichia coli* using TIRF‐M [37]. Zhang et al. investigated the development mechanism of P62/SQSTM1, a selective autophagy receptor that mediates the production of ubiquitinated proteins, using TIRF microscopy [38].

### 4.4 Super‐Resolution Microscopy Techniques

Several biological studies have utilized single‐molecule localization super‐resolution microscopy (SMLM) techniques, such as photo‐activated localization microscopy (PALM) and stochastic optical reconstruction microscopy (STORM) [39]. In PALM, a weak short‐wave laser beam randomly activates a portion of dormant fluorescent groups [32]. STORM increases the rate of super‐resolution image acquisition when compared to PALM [32]. An additional step that takes a lot of time in PALM is the photobleaching of active fluorophores [32]. Light‐switchable fluorophores, which can be activated (turned on) and deactivated (turned off) at a whim, are used by STORM to replace this step [32]. Super‐resolution microscopy techniques with their high spatial resolution allow for a detailed examination of the properties and early morphology of LLPS droplets [32].

### 4.5 Fluorescence Recovery After Photobleaching (FRAP)

Fluorescence recovery after photobleaching (FRAP) is also a commonly used technique to study LLPS. FRAP is used to measure the mobility of molecules within a condensed phase, while FCS is used to measure the exchange of molecules between two phases. In addition to these techniques, several computational approaches have been developed to study LLPS, including sequence analysis and molecular dynamics simulations. Because of the liquid diffusion phenomena, the FRAP approach enables the replacement of bleached protein molecules with unbleached ones, indicating the recovery of the fluorescence signal. FRAP experiments yielded information about the rate of tagged protein diffusion within a droplet as well as the rate of protein exchange between the droplet's exterior and interior. Around 80% of the fluorescence signals in the droplets recovered after photobleaching in around 60 s. The size of the bleached area concerning the droplet influences the recovery process if diffusion is, as predicted by LLPS, the limiting factor.

### 4.6 Fluorescence Correlation Spectroscopy (FCS)

With fluorescence correlation spectroscopy (FCS), which measures the fluorescence intensity fluctuation within a small volume owing to molecule diffusion over time to obtain detailed information about the size, dynamics, and concentrations of fluorescent particles within, it is possible to more precisely determine the diffusion of particles [40] and the molar concentration of molecules with fluorescent labels that diffuse through the confocal observation volume [41]. Using this technique, it was found that 20 nM was the threshold concentration for FUS to phase separate [42]. Plotting FUS phase diagrams based on τ_D_ (Diffusion Time) at various NaCl concentrations revealed that FUS‐EGFP phase separation was inclined to be enhanced by both high and low salt concentrations [42]. The results also demonstrated that ATP had a considerable inhibitory effect on FUS phase separation, with an IC_50_ of 3.2 mM and a relatively high inhibition effectiveness for ssDNA carrying three copies of TCCCCGT [42]. To sum up, FCS can be used for drug screening of neurodegenerative illnesses and offers a comprehensive insight into the FUS phase transition process from individual molecules to nanoscale condensates at nanomolar concentrations [42].

### 4.7 Phase Diagram

Phase diagrams characterize the conditions that lead to single‐phase or two‐phase systems in LLPS. By systematically changing variables such as concentration and salt content, researchers can determine the conditions that promote the formation of a dense phase in the solution. The phase diagram includes a coexistence line separating one‐phase and two‐phase regimes, as well as a spinodal line indicating the region where the system must undergo demixing via spinodal decomposition. The generation of phase diagrams provides insights into how the properties of molecules can affect phase separation and whether it can occur in physiological contexts. It is important to note that while phase diagrams provide valuable information, the actual processes occurring in complex cellular environments may not always be accurately represented by these simplified diagrams.

### 4.8 P‐Body Purification by FAPS

The canonical P‐body marker, GFP‐LSM14A, is stably expressed in a human epithelial cell line (HEK293) to label P‐bodies fluorescently [43]. A HEK293 cell line expressing a shortened variant of GFP‐LSM14A (GFP‐LSM14A‐D) that did not localize to P‐bodies can be used as a control to identify P‐bodies from the fluorescence of other particles [43]. Organelle‐enriched cytosolic extracts (presorted fraction) are run through a fluorescence‐activated cell sorter after cell lysis [43]. When comparing GFP‐LSM14A P‐bodies to non‐P‐body particles found in the GFP‐LSM14A‐D extract, size and fluorescence determine which is which [43]. Sorting effectively reduces non‐P‐body particles while protecting the P‐bodies of GFP‐LSM14A [43]. The sorted GFP‐LSM14A P‐bodies maintain their spherical morphology and size, but non‐P‐body organelles are reduced, as further confirmed by epifluorescence imaging [43]. A reversible crosslinker like formaldehyde can be utilized to stabilize a GFP‐biological condensate droplet, making it suitable for flow cytometry analysis and FAPS sorting [44].

### 4.9 High‐Throughput Protein Phase Separation (HiPPS)

Based on variables that could affect protein solubility, such as pH, salt, and crowding agents, which include agents known to induce LLPS, such as polyarginine (polyR), heparin, and polyuracil (polyU), 96 conditions are created [45]. The conditions are divided into seven zones and applied to a 96‐well plate [45]. The film must be inverted and adhered to a transparent, flat surface, like an acrylic board [45]. The Operetta CLS high‐content analysis equipment scans the film immediately to track protein LLPS [45]. FUS low‐complexity domain (FUS‐LC), a well‐known LLPS protein domain, is used as a standard LLPS sample [46]. Based on the density of droplets, the microscopic images in each well are graded to examine the HiPPS result and assess the LLPS ability of proteins [45]. The LLPS conditions increase with an increase in protein concentration [45]. The LLPS of FUS‐LC is effectively stimulated by PolyR [45]. Because FUS‐LC has a high tyrosine content, polyR may use π‐cation interaction to activate FUS‐LC LLPS [46]. The RNA‐helicase DDX21N‐terminal K/E‐rich domain, also known as DDX21‐NTD, showed a strong LLPS ability in the HiPPS result, especially in the zones containing monovalent and divalent salts [45]. Salts may reduce intramolecular electrostatic interactions, facilitating intermolecular interactions and protein LLPS in light of the lysine and glutamate enrichment in DDX21‐NTD [45].

## 5 Databases and Predictive Tools in LLPS Research

In recent years, significant progress has been made in the systematic cataloguing and prediction of proteins involved in liquid–liquid phase separation (LLPS). Several specialized databases have been developed to facilitate this research, including LLPSDB, PhaSePro, PhaSepDB, DrLLPS, RNAgranuleDB, HUMAN CELL MAP, and DisProt. These repositories provide comprehensive data on phase‐separating proteins, including sequence features, interaction networks, experimental validation, and associated cellular functions [47, 48, 49].

Among these, DrLLPS contains an extensive set of entries enabling large‐scale bioinformatic analyses and cross‐species comparisons. LLPSDB is a meticulously curated database of experimentally validated LLPS proteins, offering detailed annotations on experimental setups, phase behavior, and associated biomolecular information such as sequence motifs, posttranslational modifications, and nucleic acid interactions [48]. DisProt, a database focusing on intrinsically disordered proteins (IDPs), has also begun to include LLPS‐related functional annotations, albeit still limited in detail.

Of particular note is CondensateDB, a recently established and continuously updated resource that systematically catalogs biomolecular condensates and their components across various cell types and conditions. It integrates experimental and predictive data, including functional annotations and disease associations, making it a powerful tool for understanding condensate biology at the systems level. This database represents a major breakthrough by offering a unified framework for the exploration of condensates in both normal physiology and disease states.

To complement these resources, several computational tools have been developed to predict the propensity of proteins to undergo phase separation. For instance, Pi–Pi predictor evaluates the contribution of π–π interactions to LLPS, PLAAC identifies prion‐like domains, and ZipperDB predicts fibril‐forming segments potentially involved in condensate solidification [18].

In parallel, recent studies have made strides in identifying and developing small molecule modulators that either promote or inhibit phase separation. These molecules can serve as chemical probes for dissecting LLPS mechanisms and hold promise for therapeutic interventions, particularly in diseases where aberrant condensate formation plays a role. For example, compounds targeting the RNA‐binding protein FUS or modulating stress granule dynamics are currently under investigation for neurodegenerative and oncological applications.

## 6 LLPS in Subcellular Organelles

**FIGURE 3:**
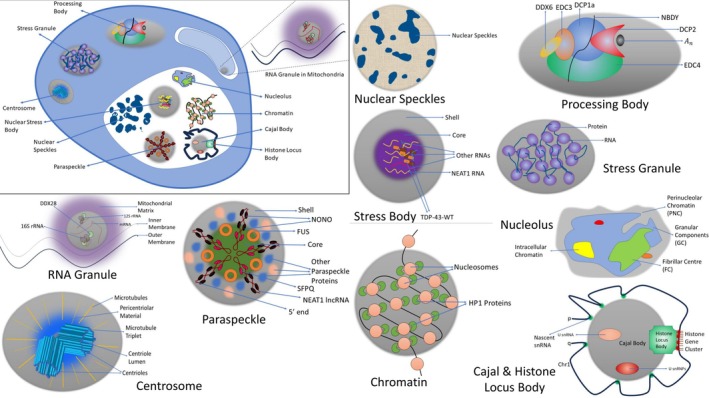
LLPS‐mediated membraneless organelle assembly: A detailed look at key protein components: The image depicts a diverse array of liquid–liquid phase‐separated membraneless organelles (MLOs) that exist within cells. The cytoplasmic MLOs include processing bodies, stress granules, and centrosomes. The mitochondria contain RNA granules, whereas the nucleus contains nuclear stress bodies, nuclear speckles, paraspeckle, nucleolus, chromatin, Cajal body, and histone locus body.

RNA‐ and protein‐containing dynamic, size‐dependent, cytoplasmic and nucleoplasmic biological condensates that go by generic names like ribonucleoprotein (RNP) granules/bodies or RNP droplets since they usually include both RNA and protein [50]. When these biological condensates fuse, they behave like liquids, dripping, soaking, and relaxing into spherical formations [51]. They exhibit a spherical shape and can be visually distinguished as micron‐sized droplets [7, 52], exhibit liquid‐like properties, distinct morphologies, and defined distribution patterns [52], with their structure regulated by a specific combination of resident proteins at the molecular level (See Figure 3A,B) [52]. The processes of protein–protein, protein–RNA, and/or protein–DNA interactions regulate and mediate their overall biogenesis [53]. The cytoplasm or nucleoplasm in direct contact with the biological condensate components characterizes the highly dynamic nature of these organelles [54]. These structures can be classified as liquid–droplet phases of the nucleoplasm or cytoplasm due to their highly dynamic internal nature [50, 52] and have only a slightly higher density compared with the surrounding cytoplasm or nucleoplasm [52, 55]. Some well‐characterized LLPS (See Figure 3) have been discussed below.

### 6.1 LLPS in Cytoplasm

#### 6.1.1 Stress Granules (SGs)

In response to stress, translation is stopped in eukaryotic cells, and following that, some cytoplasmic biological condensates and stress granules (SGs) are formed. SGs are cytoplasmic mRNPs containing several proteins that influence mRNA function in addition to translation initiation machinery proteins and nontranslating mRNAs [56]. eIF2, eIF3, Poly(A) binding protein (PABP), 40S ribosomal subunits, eIF4A, eIF4B, eIF4E, and eIF4G, as well as numerous RNA‐binding proteins that influence mRNA structure and function [52], such as HuR, Staufen, Smaug, TTP, Fragile X mental retardation protein, G3BP, CPEB, and SMN, are among the typical components of SGs [57]. SGs serve as mRNA sorting hubs, monitoring mRNP complex integrity and composition to guide them toward storage, degradation, or reinitiation pathways [52, 57]. The self‐aggregation of RNA‐binding proteins TIA‐1 and TIAR is essential for SG formation [52, 58].

#### 6.1.2 Processing Bodies or P‐Bodies (PBs)

The components of the RNA‐induced silencing complex and the mRNA decay machinery are typical components of the PB content [59]. The prototypical biomolecular condensates known as RNA processing bodies (P‐bodies) are made up of proteins and RNA that are involved in RNA metabolism, such as enzymes with RNA helicase (Dhh1), decapping (Dcp2), and exonucleolytic (Xrn1) functions [60]. It is speculated that P‐bodies act as locations for elevated RNA degradation and/or RNA/protein storage [61]. According to earlier biochemical reconstitutions, several distinct P‐body protein combinations, such as Dcp2, Edc3, Dcp1, and RNA; Dhh1, Pat1, and RNA [62]; and Dcp1, Dcp2, Lsm1‐7, Pat1, and RNA are adequate to produce condensates in vitro [63].

#### 6.1.3 Germline P‐Granules (Nuages)

Nuages are spherical, extremely dynamic biological condensates whose number, size, and composition vary throughout these organelles' existence [64]. Early spermatocytes have nuages in their cytoplasm, next to nuclei, but they travel toward the base of the flagellum before disintegration during spermatogenesis [64]. To temporarily reduce the activity of transposons, germ granules (GG) are involved in the processes of mRNA and protein sequestration and inactivation [65]. Reversible polyvalent interactions allow diffusely scattered GG components to assemble into liquid drops during liquid–liquid phase transitions or “solid” bodies during liquid–solid state transitions [65].

#### 6.1.4 Neuronal RNA Granules

The functional units for translational regulation and transport are neuronal RNA granules [66]. The granules' most prevalent mRNAs encode cytoskeletal proteins that are expressed in synaptic and neuronal projection compartments during development [67]. However, the proteomics method identified key RBPs known as nRNAg components, including Caprin‐1, FMRP, Staufen 1 or 2, G3BP1, and others [68]. Using pure nRNAg samples, chemical cross‐linking tests identified a putative G3BP2 binding site on the ribosome [69]. The distinctive morula‐like structure of ribosome‐containing RNA granules is known to be characterized by the close packing of individual ribosomes against one another [70].

#### 6.1.5 Centrosomes

The centrosome is an essential organelle of the spindle pole, with a unique architecture that contains two orthogonally organized, nine‐fold symmetrical suborganelles called mother (fully mature) and daughter (partially mature) centrioles [52]. These suborganelles are primarily made of tubulin [52]. While specific PCM proteins, including γ‐tubulin, CNN/CDK5RAP2, and Spd‐2/Cep192, are specifically recruited during mitotic entry, others, such as Plk4, Asl/Cep152, and Dplp/pericentrin, remain consistently associated with the mother centriole [52, 71]. Generally regarded as the primary hubs for organizing microtubules within the cell, these organelles also serve several other crucial roles, including assembly of the mitotic spindle, initiating the interphase microtubule array, responding to DNA damage, and regulating and controlling the efficient progression of the cell cycle [52, 72].

### 6.2 LLPS in Mitochondria and Chloroplasts

#### 6.2.1 Mitochondrial RNA Granules

The RNA granules in mitochondria are “centres for posttranscriptional RNA processing and the biogenesis of mitochondrial ribosomes,” including a wide range of proteins involved in RNA metabolism [73]. RNA helicases DHX30 and DDX28, as well as FASTKD2 and FASTKD5, members of the Fas‐activated serine–threonine kinase (FASTKD) family, are examples of RNA‐binding proteins that are present in mitochondrial RNA granules [73].

#### 6.2.2 RNA Granules in Chloroplasts

A proteomic study of chloroplast stress granules (cpSGs) identified 88 proteins, including CP29A and RIP1 (MORF 8), which have a prion‐like domain (PrLD), and several other proteins that have RNA recognition motifs [74]. The ability of protein–RNA scaffolds to assemble depends on these characteristics [75]. Additionally, ATPases, chaperones, and—interestingly—all three members of the magnesium chelatase complex and a few proteins linked to magnesium chelatase were located in cpSGs [76]. RNA splicing, ribosome maturation, ribonucleoprotein biogenesis, and RNA degradation require RNA‐binding proteins, in which phase separation and the consequent RNA granules in the chloroplast are essential [77].

#### 6.2.3 LLPS in Nucleus

##### 6.2.3.1 Nucleoli

The intrinsically disordered regions (IDRs) found in many nucleolar proteins frequently include charged domains [78]. The quantitative data observed in nucleoli support the idea that LLPS is the main force behind nucleolar formation [78]. This substructure could be a representation of several immiscible liquid phases that coexist [79]. The nucleolus's ability to store unfolded proteins and stop them from aggregating is surprisingly restricted since the system is saturable [80].

##### 6.2.3.2 Perinucleolar Compartment (PNC)

The heterogeneous nuclear ribonucleoprotein I (HNRP I) is a polypyrimidine tract‐binding protein that is present in PNC, an irregularly structured nuclear biological condensate. On the periphery of the nucleolus, the perinucleolar compartment (PNC) contains freshly generated RNA polymerase III (pol III) transcripts and RNA splicing factors, particularly PTBP1 [81]. This gave rise to the theory that the PNC is involved in the processing of pol III RNA [82]. Recent research has shown that the long noncoding RNA PNCTR can regulate the splicing patterns of RNA polymerase II transcripts by sequestering PTBP1 in the PNC [82].

##### 6.2.3.3 LLPS in Chromatin Organization and Epigenetic Regulation

Liquid–liquid phase separation (LLPS) plays a central role in chromatin architecture by organizing condensed nucleosome complexes into membraneless nuclear compartments. These chromatin condensates serve multiple functions, including genomic DNA compaction, protection from damage, and regulation of gene expression [83]. Chromatin LLPS is primarily driven by associative interactions, where histone tails, particularly the N‐terminal domains, and electrostatic forces mediate nucleosome clustering into dynamic droplets [84, 85, 86]. Modulation by linker histone H1 and the length of nucleosome linkers influences droplet stability and mimics chromatin compartmentalization seen in vivo [29].

Importantly, LLPS facilitates chromatin compartmentalization into transcriptionally active (A) and repressive (B) domains. For instance, the heterochromatin protein HP1α undergoes LLPS to form repressive chromatin domains, which correlate with gene silencing through histone H3K9me3 recognition. These LLPS‐driven compartments contribute to epigenetic regulation by selectively enriching or excluding transcription factors, chromatin remodelers, and histone‐modifying enzymes [87, 88].

Furthermore, LLPS is involved in the formation of enhancer–promoter condensates, which spatially organize cis‐regulatory elements and stabilize transcriptional hubs, allowing for efficient and robust gene activation [89]. Phase separation thus mediates both short‐ and long‐range genomic interactions, impacting chromatin topology and gene expression programs [89].

##### 6.2.3.4 LLPS and Metabolic Reprogramming

Emerging studies have revealed that cellular metabolism can profoundly influence LLPS behavior. Several metabolites—such as ATP, SAM (S‐adenosylmethionine), and NAD + —act not only as energy or cofactor sources but also as modulators of condensate dynamics. For example, fluctuations in ATP levels can alter condensate liquidity and assembly, while acetyl–CoA and SAM, which serve as donors for histone acetylation and methylation respectively, can shift the epigenetic landscape and indirectly influence LLPS‐driven chromatin structures [90].

Moreover, cancer cells undergoing metabolic reprogramming often exhibit aberrant LLPS behavior, linking oncogenic signaling and metabolite flux with condensate‐mediated gene regulation. This emerging interface between metabolism, epigenetics, and phase separation is an exciting frontier for both mechanistic studies and therapeutic development.

##### 6.2.3.5 Nuclear Speckles or Interchromatin Granule Clusters

Nuclear speckles display the distinctive liquid‐like characteristics and dynamic exchange of constituent RNA‐binding proteins and RNAs with the surrounding nucleoplasm that define phase‐separated membraneless entities [91]. The Serine/Arginine (SR) protein family of RNA‐binding proteins, known for their intrinsically disordered serine and arginine residue regions, is more prevalent in nuclear speckles [92]. Phase separation of nuclear speckles from the nucleoplasm depends critically on multivalent interactions between low‐complexity areas of SR proteins [26].

##### 6.2.3.6 Paraspeckles

The long noncoding RNA NEAT1 (nuclear paraspeckle assembly transcript 1, often referred to as MEN‐ε/β or VINC‐1) is the foundation for paraspeckles, a class of subnuclear entities [93]. The subdomains of NEAT1_2 are crucial for RNA stability, isoform switching, and paraspeckle assembly through a liquid–liquid phase separation (LLPS) mechanism, according to the functional dissection of NEAT1 [93]. As molecular sponges, paraspeckles interact with chromatin regions that are rich in active promoters and enhancers while also sequestering certain RNA transcripts and proteins [94].

##### 6.2.3.7 Nuclear Stress Bodies (nSBs)

Subnuclear organelles known as nuclear stress bodies (nSBs) develop in response to heat shock [95]. Through a variety of mechanisms, such as chromatin remodeling and the trapping of transcription and splicing factors, they are believed to be involved in the quick, temporary, and global reprogramming of gene expression [95]. The 141 proteins found in nSBs are primarily RNA‐binding proteins that are involved in mRNA splicing, processing, and export [96]. Two different sets of nSBs exist [97].

##### 6.2.3.8 Histone Locus Bodies (HLBs)

The transcription, histone mRNA processing, and histone biosynthesis are all regulated by the HLB, an evolutionarily conserved nuclear body [98]. Histone genes initiate the phase separation process that is controlled by cyclin‐dependent kinase (Cdk) activity to produce the HLB [99]. Presently, a big, largely unstructured Cyclin E/Cdk2 substrate protein—known as Mxc in Drosophila and NPAT in humans—found at the promoters of all human RD histone genes serves as the primary characteristic of the HLB [100].

##### 6.2.3.9 Cajal Bodies (CBs)

CBs are subnuclear structures that are frequently found in association with the nucleolus and exist in eukaryotes as 0.2 to 2‐μm particles [101]. They have been shown to play well‐defined roles in RNA metabolism and the formation of ribonucleoprotein particles (RNP) that are involved in transcription, splicing, ribosome biogenesis, and telomere maintenance [101]. The coilin protein is necessary for the formation of CBs, but so are the synthesis, processing, recruitment, and maturation of different RNAs [102]. Several snRNAs (U1, U2, U4, and U5 snRNAs) move to CBs during synthesis, and CBs play a role in the early phases of snRNP formation, too [103].

##### 6.2.3.10 PML Nuclear Bodies or Nuclear Dots (PODs)

Promyelocytic leukemia (PML) nuclear bodies (NBs) (also known as ND10) are membraneless organelles that concentrate proteins at specific locations within the nucleoplasm [104]. Most mammalian cell nuclei consist of them, and they form a sphere with a diameter of around 0.1 to 1 μm [10]. The disarray of PML NBs in acute promyelocytic leukemia (APL) led to their discovery [105]. PML, the primary organizer of PML NBs, is a multivalent protein with several modular domains and interaction motifs, an essential characteristic for polymerization‐driven liquid–liquid phase separation [105].

##### 6.2.3.11 Sam68 Nuclear Bodies (SNBs)

The SAM68 nuclear bodies are found on the surface of nucleoli [52, 106]. These entities are between one and ten in number per nucleus and range in size from 0.25 to 1.0 μm in diameter [106]. The primary constituents of the nuclear bodies of SAM68 belong to a class of RNA‐binding proteins called the signal transduction and activation of RNA (STAR) domain, which includes the GSG domain (GRP33, Sam68, and GLD‐1) [52, 106]. Sam68 plays a role in several posttranscriptional regulatory processes, including RNA export and alternative splicing [107].

##### 6.2.3.12 Cleavage Bodies

Cleavage bodies are nuclear suborganelles that include the Cleavage and Polyadenylation Specificity Factor 100 (CPSF‐100) and the Cleavage Stimulation Factor 64 (CstF‐64) [108]. Different cell cycles had different spatial distributions of cleavage bodies; cleavage bodies containing CstF‐64 were primarily enriched in the S phase, whereas cleavage bodies carrying CPSF‐100 were enriched in both the S and G2 phases [108]. A further confirmation that cleavage bodies play a role in DNA replication rather than RNA transcription would be the elimination of most CstF‐64‐containing cleavage bodies through suppression of DNA replication in cells treated with hydroxyurea [108].

##### 6.2.3.13 DDX1 Bodies

The primary function of the DDX1 body is the posttranscriptional processing of RNA [109]. Conventional biochemical experiments or mass spectrometry revealed that many proteins colocalized with MLOs by immunofluorescence or co‐complexed with known MLO scaffolds [110]. For instance, the DEAD‐box helicase DDX1, a known scaffold of numerous MLOs, including the DDX1 body, forms an RNA transport complex with the putative stress granule proteins C14ORF166, FAM98A/B, and RNA 2′,3′‐cyclic phosphate and 5′‐OH ligase (RTCB) [111].

##### 6.2.3.14 Nuclear Gems or Gemini of Coiled Bodies

High quantities of survival motor neuron (SMN) protein, the protein implicated in spinal muscular atrophy (SMA), as well as specific SMN‐interacting proteins, including Gemins, are seen in Gems [112]. Moreover, GEMs participate in transcription, translation, and spliceosomal snRNP formation [103].

##### 6.2.3.15 Oct1/PTF/Transcription Domains (OPT Domains)

OPT domains appear to function similarly to nucleoli, arranging specific genes on specific chromosomes in a way that concentrates the necessary transcription, and processing components [95]. Subsequent research revealed that the OPT domains contain a mediator of DNA damage checkpoint 1 (MDC1), phosphorylated H2AX (γH2AX), and p53 binding protein 1 (53BP1) [113]. It was also discovered that replication stress can cause biological condensates in G1 cells [113].

##### 6.2.3.16 PcG Bodies (Subnuclear Organelles Containing Polycomb Group Proteins)

Condensed structures known as Polycomb bodies are the localization of the Polycomb group (PcG) complex PRC1 in the nucleus. A critical component of epigenetic inheritance is PcG proteins. By suppressing developmental genes, particularly through the repressive mark H3K27me3 and chromatin compaction, they guarantee the preservation of cell identity. It has also been demonstrated that PcG proteins are inherited via DNA replication.

### 6.3 Polymorphic Interphase Karyosomal Association (PIKA)

Six groups comprise the PIKA classification. In this process, the smaller PIKAs from Groups 3 and 4 emerge during early S phase, even Smaller Group 5 PIKAs appear in mid‐S phase, and the Tiniest Type 6 PIKAs are detected in late S phase [52]. The larger PIKAs from Groups 1 and 2 are observed only during the G1 and early S phases [52, 114].

## 7 Physiological Functions of LLPS

**FIGURE 4:**
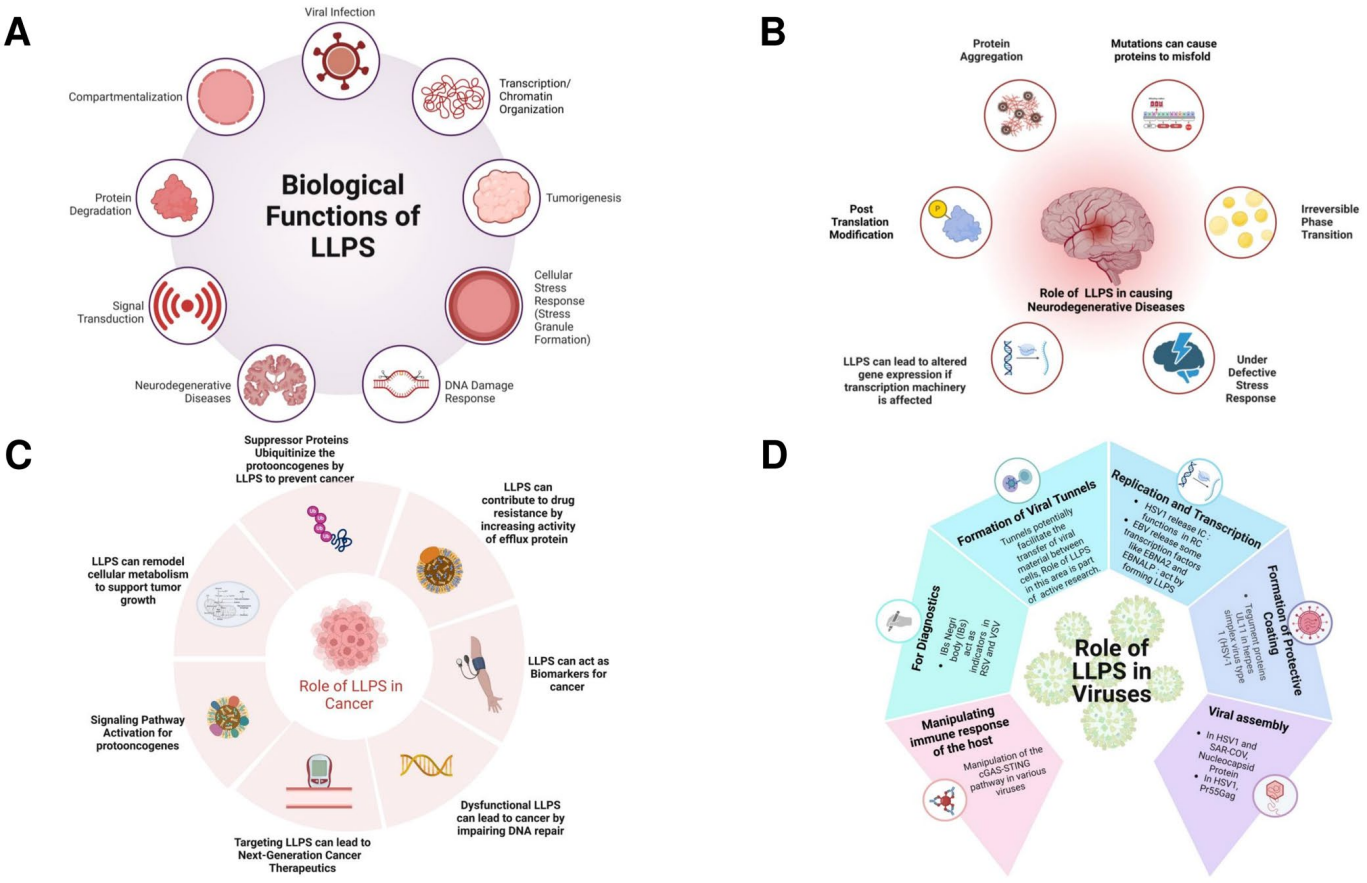
LLPS: A dynamic force in cellular biology and disease: (A) The multifaceted roles of liquid–liquid phase separation (LLPS) in cellular processes. (B) The multifaceted roles of liquid–liquid phase separation (LLPS) in neurodegenerative diseases. (C) The multifaceted roles of liquid–liquid phase separation (LLPS) in cancer. (D) The multifaceted roles of liquid–liquid phase separation (LLPS) in viruses.

Liquid–liquid phase separation (LLPS) plays a crucial role in various cellular processes, including higher‐order chromatin organization, gene expression, triage of misfolded or unwanted proteins for autophagic degradation, assembly of signaling clusters, actin‐ and microtubule‐based cytoskeletal networks, asymmetric segregations of cell fate determinants, and formation of pre‐ and postsynaptic density signaling assemblies (See Figure 4A) [19].

LLPS forms membraneless organelles in higher‐order chromatin organization that regulate gene expression by sequestering transcription factors and RNA polymerases. Recent studies point out that the cellular nucleus organization, which is composed mainly of the chromatin domain, is made of chromosomal territories and the interchromatin compartment (IC) [115, 116]. These areas are suggested to be formed by phase separation. LLPS also affects chromatin distribution in the nucleus by constraining its interactions with nuclear bodies that LLPS also forms.

The current understanding of 3D genome organization emphasizes the importance of spatial interactions between distant enhancers and promoters to activate transcription, highlighting the significance of 3D genome interactions [117, 118, 119]. Euchromatin and heterochromatin, representing active and repressed chromatin regions, display unique folding patterns within the 3D genome [120]. Through high‐throughput chromosome conformation capture (Hi‐C) analysis, it has been demonstrated that active and repressed chromatin domains segregate into A and B compartments, respectively, with topologically associating domains (TADs) folding the chromatin chain [121]. TADs are thought to form via active DNA loop extrusion. At the same time, compartmental domains appear to be a result of nucleosome condensation with specific epigenetic marks, involving liquid–liquid phase separation (LLPS) [19, 122, 123].

In gene expression regulation, LLPS is involved in the formation of transcriptional condensates that facilitate the assembly of RNA polymerase II and other transcriptional machinery [124]. The process of liquid–liquid phase separation (LLPS) is significant in regulating gene expression by promoting the formation of liquid condensates at gene regions during transcription [125]. This interaction may be aided by the interplay between newly synthesized RNA molecules and particular proteins, such as components of the splicing machinery (101). The movement of RNA polymerase II (Pol II) between different condensates is expected to be controlled by specific phosphorylation patterns on the C‐terminal domain (CTD), which can affect the presence of splicing factor condensate elements. These discoveries suggest that phosphorylated CTD operates as a framework for assembling complexes of proteins involved in transcription elongation, underscoring the role of LLPS in governing gene expression mechanisms [126, 127].

In the triage of misfolded or unwanted proteins for autophagic degradation, LLPS forms protein aggregates targeted for autophagy. In the assembly of signaling clusters and actin‐ and microtubule‐based cytoskeletal networks, LLPS is also involved in the formation of membraneless organelles that sequester signaling molecules and cytoskeletal proteins [1, 19].

In asymmetric segregations of cell fate determinants, LLPS forms membraneless organelles that sequester cell fate determinants and facilitate their asymmetric segregation during cell division.

In forming pre‐ and postsynaptic density signaling assemblies, LLPS is involved in forming membraneless organelles that isolate synaptic proteins and facilitate their assembly into pre‐ and postsynaptic density signaling assemblies. In an in vitro cell‐free reaction, LLPS suggested the design of RNA granules [1]. Many other critical biological processes, including transcription, chromatin organization, X‐chromosome inactivation (XCI), DNA damage response (DDR), tumorigenesis, and autophagy, have been shown to utilize LLPS to generate the relevant membraneless condensates and achieve their specific functions [26].

## 8 Role of LLPS in Human Diseases

Liquid–liquid phase separation (LLPS) has significantly impacted our knowledge of biological processes and diseases. Studies have demonstrated LLPS's crucial role in physiology and disease, affecting immune responses, genome organization, and neurodegenerative conditions such as ALS, FTD, AD, and PD (See Figure 4B). The formation of pathological protein aggregates in these illnesses is linked to abnormal phase separation, shifting from reversible LLPS to irreversible aggregation due to mutations and posttranslational changes. LLPS also presents new therapeutic possibilities by targeting disease‐related proteins, as evidenced by the effect of SHP2 mutants and SRC‐1 on cancer. The LLPS field is continuously progressing, offering promising advancements in clinical approaches and therapeutic strategies.

### 8.1 LLPS in Neurodegenerative Diseases

The comprehension of neurodegenerative disorders has long been a complex task. Still, with the emergence of the liquid–liquid phase separation (LLPS) concept, researchers now believe that pathological aggregates resulting from numerous abnormal protein accumulations in the body may contribute to developing certain neurodegenerative conditions [1]. These aggregates stem from reversible dynamic LLPS transitioning into irreversible clusters, such as α‐synuclein in Parkinson's disease (PD), tau in Alzheimer's disease (AD), fused in sarcoma (FUS), and TAR DNA‐binding protein 43 (TDP‐43) in amyotrophic lateral sclerosis (ALS) [128]. Disease‐associated mutations and posttranscriptional modifications are thought to trigger these transitions [129]. As protein aggregates accumulate in various brain regions, they affect multiple brain functions and can result in neurodegenerative illnesses. The interaction of RNA‐binding proteins (RBPs) within the separated phase also facilitates the formation of abnormal aggregates by promoting the transformation of RBPs into solid aberrant clusters.

TDP‐43, an RNA‐binding protein crucial for RNA processing, can aggregate in cytoplasmic neuronal inclusions through its C‐terminal domain (CTD) in Amyotrophic Lateral Sclerosis (ALS) [130]. TDP‐43's LLPS is mediated by aromatic, dipolar, positively charged (arginine) residues in the intrinsically disordered region (IDR) and a transiently formed α‐helix. Most ALS‐linked helix mutations decrease LLPS, with the unusual exception of A321V, which increases it. TDP‐43's LLPS formation is also partially mediated through the N‐terminal domain (NTD) when it forms linear chains, promoting oligomerization. In contrast, introducing a phosphomimic substitution at position S48 hinders this polymeric assembly. This disruption discourages liquid–liquid phase separation (LLPS) in laboratory settings, affects the behavior of liquid–liquid phase‐separated nuclear TDP‐43 constructs in cells, and impairs RNA splicing function [131].

FUS's function in ribonucleoprotein granules and other membrane‐free organelles involves reversible phase separation, primarily influenced by its intrinsically disordered low‐complexity (LC) domain. Cation–π interactions between tyrosine in the LC domain and arginine in structured C‐terminal domains play a role in this process. Posttranslational arginine methylation can modulate these interactions, with arginine hypomethylation enhancing phase separation. If substantial hypomethylation exists, FUS condensates are converted to stable intermolecular β‐sheets. This rich hydrogel interferes with the RNP‐granule function of protein formation in the neuronal terminals, leading to FUS‐associated frontotemporal lobar degeneration (FTLD). As the interaction between the arginine–glycine‐rich motif's arginine (positively charged) and tyrosine (aromatic) in the IDR of FUS increases, condensate takes place. LLPS decreases when multiple phosphorylation and methylation events occur at various positions along the FUS sequence [132, 133].

Studies have demonstrated that α‐Synuclein droplets with liquid‐like properties created in laboratory settings eventually solidify, forming an amyloid hydrogel composed of oligomers and fibrillar structures [134]. Factors that accelerate α‐Synuclein aggregation, including low pH, phosphomimetic substitutions, and mutations linked to familial Parkinson's disease, also promote phase separation into liquid states and hydrogel formation [134]. The research revealed that α‐Synuclein liquid–liquid phase separation (LLPS) is affected by electrostatic interactions in the N‐terminal amphiphilic region and hydrophobic interactions in the non‐Ab component of AD plaque (NAC) domains. Additionally, Parkinson's disease‐associated mutations (E46K, A53T) and S129 phosphorylation enhance LLPS [135].

According to recent research, tau, a protein implicated in neurodegenerative diseases such as Alzheimer's disease, is prone to liquid–liquid phase separation (LLPS). Hydrophobic interactions play a surprisingly minor role in tau LLPS, while intermolecular electrostatic interactions between the positively charged middle/C‐terminal and negatively charged N‐terminal regions drive the majority of the process [136]. It has been demonstrated that truncation, mutation, and hyperphosphorylation increase tau LLPS and aggregation. p300 histone acetyltransferase (HAT) hyperacetylates tau to the detriment of LLPS, prevents heparin‐induced aggregation, and obstructs access to LLPS‐initiated microtubule assembly [134]. It is estimated that even though tau acetylation inhibits the harmful effects of LLPS‐dependent aggregation, it still plays a role in the pathophysiology associated with tau loss of function by impeding tau LLPS‐mediated microtubule assembly [134]. Also, phosphorylating residues S199 and S202, as well as introducing a mutation in P301L, have been demonstrated to enhance liquid–liquid phase separation (LLPS) [137].

Changes in protein phase separation caused by mutations can result in permanent aggregation. Mutations that impact binding to cellular regulators can disturb normal physiological processes. Abnormal transitions may be initiated by posttranslational modifications. More investigation is required to comprehend and potentially create novel medications that target these processes [1].

### 8.2 LLPS in Cancer

Speckle‐type POZ protein (SPOP), a tumor‐suppressor protein, can form many solid tumors, including prostate, gastric, and colorectal cancers, when it is mutated somehow. Using the ubiquitin–proteasome system, SPOP facilitates the degradation of a cullin3‐RING ubiquitin ligase's substrates by acting as a substrate adaptor. Protooncogenic proteins are the substrates of SPOP. These proteins can accumulate and change susceptible cell types in an oncogenic manner [138]. These substrates are colonized and phase‐separated with SPOP to create condensates. The condensates of SPOP facilitate the ubiquitination of these substrates. The components of SPOP include two dimerization domains, BTB and BACK, and a substrate‐binding Meprin and TRAF Homology (MATH) domain. Phase separation of SPOP requires the self‐association of two dimerization domains and the multivalent contacts between the MATH domain and substrates [139]. It is observed that there is an accumulation of SPOP substrates into the protein aggregates due to a cancer‐associated mutation in the MATH domain. When the MATH domain is mutated, it hinders the formation of proper SPOP condensate as the substrates cannot interact with the SPOP. These accumulated substrates lead to cancer [140].

PTPN11 encodes the Protein Tyrosine Phosphatase nonreceptor type 11 (PTP)/Src homology region 2 domain‐containing phosphatase‐2 (SHP‐2), which is a protooncogene, that is, if this gene is mutated, it will become cancerous. This gene is crucial for signaling Ras‐mitogen‐activated protein kinase (MAPK) during normal development. When the signal is received, SHP2 in the cytoplasm detects it and opens, taking part in intracellular signaling as the signal decreases and begins to close again. A group of disease‐associated SHP2 mutations share liquid–liquid phase separation (LLPS) characteristics. The conserved well‐folded PTP domain mediates SHP2 LLPS through multivalent electrostatic interactions, and an auto‐inhibitory mechanism regulates it. SHP2 PTP activity is increased when the SHP2 mutant's LLPS is attenuated by SHP2 allosteric inhibitors by locking it in a closed conformation. Furthermore, wild‐type (WT) SHP2 can be attracted to and activated by disease‐associated SHP2 mutations in LLPS to facilitate MAPK activation. These findings not only imply that LLPS plays a gain‐of‐function role in the pathophysiology of human disorders linked to SHP2, but they also offer proof that PTP may be controlled by LLPS [141].

AKAP95 is also a nuclear protein that plays a role in supporting tumorigenesis by regulating splicing and forming phase‐separated condensates. Studies have shown that changing specific amino acids in AKAP95 can disrupt its condensation in either a positive or negative way. The function of AKAP95 in regulating splicing is lost when condensation is disrupted or compromised, when condensates become rigid. If it is restored by replacing its condensation‐promoting area with similar regions from other proteins, additionally, the roles of AKAP95 in controlling gene expression and promoting tumorigenesis depend on its ability to form dynamic and fluid condensates. These findings establish a connection between phase separation and tumorigenesis (See Figure 4C). This can help in making therapeutics and preventing the growth of cancer [142].

Mutation and dysregulation of transcription can cause cancer. Certain transcriptional coactivators, such as Yes‐associated protein (YAP) and transcriptional coactivators with PDZ‐binding motif (TAZ), undergo LLPS and are involved in tumorigenesis [143]. In healthy cells, the Hippo pathway is activated by various signals, suppressing YAP and TAZ activity, and inhibiting the expression of genes regulating cell growth and tumorigenesis [144]. However, the Hippo pathway in cancer cells is often deactivated, accumulating YAP and TAZ, and promoting cancer cell proliferation [145].

LLPS regulates the transcriptional activity of YAP/TAZ in cancer cells, driving cell proliferation and resistance to anti‐PD‐1 immunotherapy [146]. YAP condensates are enriched with accessible chromatin domains, specifically super‐enhancers, while TAZ condensates contain TEAD4 (DNA‐binding cofactor), BRD4, MED1, and CDK9 (cofactors). The intrinsically disordered TA and CC domains are crucial for YAP condensate formation. The coiled‐coil (CC) and WW domains are essential for TAZ phase separation. Nuclear condensate formation of YAP and TAZ, controlled by Hippo signaling, phosphorylates the coactivators through large tumor–suppressor kinases (LATS), thereby negatively regulating their LLPS.

A dysregulated gene transcription disorder, or gene mutation, is also thought to cause cancer. Protooncogene mutations usually result in more gene products or increased activity of the products, which stimulates uncontrollably high cell proliferation to produce tumors. Likewise, decreased biological activity of mutant cancer suppressor proteins may encourage the growth of malignancies.

### 8.3 LLPS in Viral Infection

The virus is a submicroscopic infectious agent in the environment in a dormant form and gets activated in contact with living organisms. They are highly dependent on host organisms to replicate. Viruses reconstruct the cellular components and utilize the biomolecules for multiple functions like replication, assembly, and packaging. It is believed that viruses use the biomolecular agglutinates, which eventually give rise to condensates due to an increase in local concentration and contact within the molecules (See Figure 4D). This is how viruses evolved to use the host's biomolecules to produce LLPS and make them function for themselves [147].

### 8.4 The Roles of Phase Separation in Virus Replication and Transcription

Viruses like the Herpes virus family (DNA viruses) use phase separation for replication and transcription. Herpes Simplex Virus Type 1 (HSV‐1) enters the nucleus of host cells to perform these functions by releasing some immediate‐early, early, and late proteins [147]. One of the immediate‐early proteins, ICP4, is an intrinsically disordered protein released by HSV‐1, which contributes to the formation of condensates via LLPS [147, 148]. It was studied that ICP4 is localized to the replication compartment (RC); hence, it is believed that RCs may be a product of phase separation [149]. Tegument proteins in Herpes Simplex Virus Type 1 (HSV‐1) serve as connectors between the capsid and viral envelope [147], aiding in the formation of a protective coating. A recent in vitro study has shown that a specific tegument protein, UL11, contains an intrinsically disordered region capable of forming LLPS [150]. Many other tegument proteins in HSV‐1 are also seen to share these characteristics and hint at a potential role for these proteins in creating the tegument assembly via LLPS.

Epstein–Barr virus (EBV) is from the gamma subfamily of the Herpesviridae family, which is related to multiple malignant tumors, including nasopharyngeal carcinoma and gastric cancer [151]. These viruses release some transcription factors like EBNA2 and EBNALP, which are released in an early infective phase within B cells and can form condensate via LLPS [152, 153]. Epstein–Barr virus nuclear antigen 2 (EBNA2) has two terminals; the N‐terminal induces accessible chromatin domains (ACDs) within the host and undergoes phase separation, whereas its acetylation is done by recruiting histone acetyltransferase p300 via the C‐terminal [154]. This resultant phase separation supports epigenetic regulation of chromatin activation and genomic transcription by remodeling of chromatin topology [147].

Another role of LLPS in viruses is seen via inclusion bodies (IB), the membraneless organelles formed by LLPS that play vital roles in the replication of RNA viruses from the family of nonsegmented negative‐strand (NNS) RNA viruses, for example, in Respiratory Syncytial Virus (RSV). Here, IBs are formed when the C and N termini of the phosphoprotein (P) protein interact with the nucleocapsid (N) protein's N and C termini, respectively [155, 156]. The presence of N and P proteins alone can initiate the formation of a small Negri body (NB). NBs are spherical structures that can fuse and temporarily alter their form by deforming when confronted with a physical barrier. However, the creation of viral inclusion bodies (IBs) in Vesicular stomatitis virus (VSV) requires the involvement of not only N and P proteins but also the Large (L) protein, which has the role of RNA‐dependent RNA Polymerase [157].

### 8.5 Role of LLPS in Viral Assembly

HIV‐1 has a complex lifecycle; it undergoes fusion, decapsulation, reverse transcription, and integration into the host cell, resulting in the formation of a provirus. Additionally, a substantial amount of positive‐stranded RNA is transcribed before exiting the cell nucleus. These RNAs have a dual function, that is, they can act as mRNA, initiating the synthesis of viral proteins as well as vRNA for forming immature viral particles that bud and mature [158]. Nucleocapsid‐mediated LLPS regulates this balance between mRNA translation, RNA, and packaging protein [147, 159].

Pr55Gag, referred to as the HIV‐1 Gag polyprotein precursor, controls the assembly of the virus. The p6 structural domain, two spacer peptides, SP1 and SP2, capsid (CA), nucleocapsid (NC), and several monomeric protein matrices (MA) are separated from the gag by proteolytic shearing [160]. Important cycle activities like vRNA capsulation and Gag multimerization depend on NC, which can form condensates via LLPS. LLPS is responsible for translational silencing, and it also affects the balance among vRNA and NC‐mediated stress granules, resulting in the formation of viral particles. The nucleocapsid protein of SARS‐CoV‐2 condenses into liquid‐like organelles, which help in packaging viral RNA and evading host immune responses [161].

### 8.6 Role of LLPS in the Virus for Evading Host Immunity

Viruses can also induce the innate immunity of a host by engaging the immunity‐activating molecule in the droplet formulation. It is seen that LLPS [162] regulates the cGAS–STING immune pathway. The vDNA activates cGAS, therefore producing the second‐messenger molecule called 2′3′‐cGAMP, which gets activated when vDNA is recognized by the STING protein on the endoplasmic reticulum. cGAMP binding to STING leads to conformational changes in STING, hence activating the TBK1‐IRF3 pathway, which produces type 1 IFN [163, 164]. IFN is responsible for signaling the immune system of the host. Viruses prevent this by forming LLPS in 2 ways. One way of forming LLPS is by promoting the binding of the nonfixed N‐terminal and nucleotidyl transferase (NTase) domain on the C‐terminal of cGAS to dsDNA through multivalent interactions [165]. Another way is when excess 2′3′‐cGAMP, STING, and unphosphorylated TBK1 form LLPS and inhibit the phosphorylation of IRF3, leading to the prevention of overstimulation of the cGAS–STING pathway, which does not allow host cells to signal the immune system by blocking IFN production [147, 166, 167].

## 9 Therapeutic and Diagnostic Applications of LLPS

The discovery of mechanisms involved in the progression of novel diseases has led to the development of new therapeutic techniques. LLPS, being an integral part of pathogenesis for various diseases, makes it a potentially effective therapeutic target. As previously mentioned, the normal physiological state can be altered by the formation (as in some cancers and neurodegenerative diseases) or disruption (as in developmental disorders or regulatory condensates) of biomolecular condensates, leading to diverse pathological and physiological conditions. Targeting the key regulators of these condensates offers an unprecedented opportunity to treat once‐incurable conditions.

One conventional approach to target the LLPS is via targeting the scaffold proteins responsible for LLPS formation. This can be achieved primarily by subjecting the proteins to posttranslational modifications or by inhibiting the key enzymes responsible for LLPS formation or disruption [168]. Examples of PTMs modulating LLPS include the phosphomimetic mutation of TDP‐43, which decreases LLPS formation [131], and the phosphorylation of TAU [33], FMRP, and CAPRIN1 [169], which promote LLPS formation.

Small molecules have also emerged as effective modulators of biomolecular condensates. In 2021, Sergey V. Ulianov et al. demonstrated that 1,6‐hexanediol (1,6‐HD) played an important role in disrupting LLPS and altering chromatin organization, leading to enlarged nucleosome clusters, partial mixing of A/B compartments, and weakened enhancer‐promoter interactions. These changes were largely irreversible, suggesting that LLPS plays a constrained but essential role in maintaining higher‐order chromatin structure and compartmentalization by compromising the 3‐D genome organization in living cells and inhibiting LLPS [170, 171]. JQ1, a BET bromodomain inhibitor, disrupts super‐enhancer‐associated condensates, thereby inhibiting the transcriptional programs crucial for survival of myeloma and ovarian cancer cells [172, 173]. Small molecules that preferentially bind proteins in either the soluble or condensed phase can shift the equilibrium, thus modulating LLPS dynamics. Lipoamide, for instance, targets the FUS protein, which is significantly implicated in the progression of ALS and FTD [174]. Lipoamide is a small molecule capable of modulating LLPS and stress granule dynamics, dissolving cytoplasmic stress granules formed by mutant FUS and TDP‐43. This action allows these proteins to relocalize to the nucleus, restoring their nuclear activity, including DNA repair and RNA splicing [175].

In hepatocellular carcinoma (HCC), LLPS influences crucial cellular processes in both the cytoplasm and nucleus, such as signaling pathways (e.g., MAPK, cAMP‐dependent, Hippo), gene expression, RNA dynamics, autophagy, and ferroptosis. Specific long noncoding RNAs (e.g., FASA and URB1‐AS1) regulate ferroptosis and iron homeostasis via LLPS, impacting cancer progression and treatment outcomes. To address these, scientists have introduced LLPS‐related gene modules and the LLPSRI, a promising prognostic marker for HCC, as predictive tools. They identified 43 LLPS‐related genes associated with survival and proposed CRISPR‐Cas9, RNAi, PROTACs, and small molecules (e.g., Sorafenib, Elvitegravir) as potential interventions [176]. Numerous other small molecules are known to target LLPS [171, 177, 178]. Industries, including Dewpoint Therapeutics and Nereid Therapeutics, are actively pursuing approaches involving small molecules and shape‐shifting nanoparticles to target LLPS.

Gene‐editing tools like CRISPR‐Cas9 and RNAi can target genes encoding intrinsically disordered regions (IDRs) that facilitate LLPS, thereby modulating condensate dynamics and affecting cancer cell survival. Recently, scientists have refined the application of these genetic tools to modulate LLPS activity. CRISPR‐based transcriptional activation (CRISPRa) systems, such as DropCRISPRa, leverage LLPS to enhance gene activation. These systems fuse CRISPR‐associated proteins (e.g., dCas9) with IDRs from proteins like FUS or TAF15. This fusion leads to the creation of phase‐separated condensates that efficiently recruit transcriptional machinery (e.g., RNA polymerase II, BRD4). This method has successfully achieved robust gene activation in mammalian cells and mice, underscoring its potential for synthetic biology and therapeutic applications [179].

Traditional strategies such as proteolysis‐targeting chimaeras (PROTACs) can also be employed to target proteins that drive LLPS. PROTACs induce the degradation of tagged proteins by recruiting them to the ubiquitin–proteasome system, thereby preventing condensate formation [180]. While this method necessitates extensive knowledge of specific pathogenic condensate formation pathways, it holds promise as a highly effective strategy for treating rare diseases and combating pathogens that utilize LLPS as a mechanism. Furthermore, scientists have developed a more advanced application of LLPS for targeted protein degradation: Phase separation‐enhanced PROTAC (PSETAC). PSETACs are constructed with an intrinsically disordered region positioned between the target protein ligand and the proteasome degradation tag. Upon deployment, this IDR promotes LLPS formation around the target, which enhances the efficiency of recruited proteasomes in degrading the target protein [181].

Scientists have also devised innovative ways to utilize LLPS as a diagnostic tool. A recent study introduced a simple, label‐free method for detecting specific nucleic acid sequences using CRISPR‐Cas12a and Cas13a enzymes, which recognize DNA and RNA targets, respectively. Upon target detection, these enzymes trigger nonspecific nucleic acid cleavage, which in turn induces LLPS in solutions containing charged polymers. The formation of LLPS results in visible turbidity, allowing for naked‐eye detection. This approach is based on the Voorn‐Overbeek model, which predicts the occurrence of LLPS due to the extension of the polymer to a certain length. This method offers a cost‐effective and visually accessible alternative to traditional CRISPR‐based molecular diagnostics, paving the way for widespread and rapid diagnostic applications [182]. Further comprehensive research on this subject is essential to unlock its full potential and drive significant advancements in the field of science.

## 10 Conclusion

In essence, liquid–liquid phase separation (LLPS) has fundamentally reshaped our understanding of cellular organization and function. This phenomenon, driving the formation of numerous membraneless organelles, plays a pivotal role in gene expression, development, and disease progression. It provides a robust framework for deciphering the intricate spatial organization of the cell, particularly within the nucleus, where it underpins essential genome activities. Numerous membraneless organelles are present across the cell, which are formed by LLPS and have significance in basic cellular mechanisms.

The dynamic interplay of proteins, RNA, and environmental factors governs the formation and stability of LLPS condensates. However, disruptions in this delicate balance can lead to cellular dysfunctions and contribute to the pathogenesis of diseases like neurodegenerative disorders and cancer. Consequently, elucidating the molecular mechanisms of LLPS and its dysregulation is critical for developing innovative therapeutic interventions.

While advancements in optical microscopy methodologies, fluorescence methods, and computational modeling have significantly enhanced our ability to study LLPS, further research is imperative to unravel its complexities fully. The databases and predictors have helped to analyze and record the protein characteristics crucial to the LLPS study. A deeper understanding of the intricate relationship between LLPS and cellular pathophysiology is essential for advancing our knowledge of human health and disease. Ultimately, LLPS stands as a central organizing principle within the cell, and its continued exploration promises to yield transformative insights with significant implications for both basic biology and clinical applications.

## Author Contributions

Dikesh Kumar Thakur and Sonal Padole gathered information, wrote the manuscript, and prepared the figures. Tapati Sarkar and Somasundaram Arumugam wrote and edited the manuscript. Shiladitya Chattopadhyay conceptualized the paper, wrote and edited the manuscript, and finalized the figures.

## Conflicts of Interest

The authors declare no conflicts of interest.

## Data Availability

Data sharing does not apply to this article as no datasets were generated or analyzed during the current study.
